# Obesity and Its Association With Micronutrient Deficiency Among Mexican Children and Adolescents: A Systematic Review and Meta-analysis

**DOI:** 10.1093/nutrit/nuaf148

**Published:** 2025-08-13

**Authors:** Alejandra Herrera-González, Yareni Yunuen Gutierrez-Gómez, Carlos Francisco Moreno-García, Magaly Aceves-Martins

**Affiliations:** The Rowett Institute of Nutrition and Health, University of Aberdeen, Aberdeen AB25 2ZD, United Kingdom; School of Medicine and Health Sciences, Tecnologico de Monterrey, Monterrey 64700, Mexico; School of Medicine and Health Sciences, Tecnologico de Monterrey, Guadalajara 45201, Mexico; School of Computing, Robert Gordon University, Aberdeen AB10 7QB, United Kingdom; The Rowett Institute of Nutrition and Health, University of Aberdeen, Aberdeen AB25 2ZD, United Kingdom

**Keywords:** obesity, children, adolescents, Mexico, vitamins, minerals

## Abstract

**Context:**

Obesity and micronutrient deficiencies in the pediatric population of Mexico pose significant public health challenges. However, the relationship between these two conditions is still being studied.

**Objective:**

To systematically review evidence on the association between overweight and obesity and micronutrient deficiencies among Mexican children and adolescents.

**Data Sources:**

A systematic search was conducted in 13 databases and one search engine.

**Data Analysis:**

Sixteen studies met the eligibility criteria and were included in this review. A total of 20 043 participants were included across the included studies, and the results were highly varied, as not all micronutrients showed a significant deficit in the presence of obesity. Calcium, phosphorus, magnesium, and vitamin E deficiencies were noted among participants with obesity; however, these did not significantly differ from those of individuals with normal weight. Current evidence suggests that Mexican children who have overweight or obesity are more likely to have zinc, iron, and vitamins D and B_6_ deficiency. A random-effects meta-analysis of 4 studies showed that children who have overweight or obesity were significantly more likely to have a deficit of vitamin D (odds ratio [OR], 1.84; 95% CI, 1.46–2.32), which was stronger for school-aged children (OR, 1.99; 95% CI, 1.56–2.55).

**Conclusion:**

Current evidence suggests that Mexican children and adolescents who have overweight or obesity are more likely to experience vitamin D deficiency, and some evidence suggests that they are also prone to zinc, iron, and vitamin B_6_ deficiencies. For this reason, health-promotion and -prevention efforts must be comprehensive and address micronutrient deficiencies, common risk factors, and broader social determinants linked to noncommunicable diseases.

**Systematic Review Registration:**

PROSPERO registration no. CRD42019154132.

## INTRODUCTION

Globally, the prevalence of overweight and obesity has been increasing, and Mexico is no exception. The Mexican National Survey of Health and Nutrition (ENSANUT), a nationally representative probabilistic survey conducted to monitor health and nutrition trends, reported that, during the period from 2020 to 2022, 37% of schoolchildren and 41% of adolescents were found to have either overweight or obesity. Percentages have been rising for the last couple of decades; there was a 24% increase (3.5 percentage points) in obesity prevalence between 2006 and 2020–2022.^1^^,^^2^

Factors contributing to obesity among Mexican children and adolescents include the obesogenic environment they grow up in, socioeconomic influences,^3^ and cultural aspects.^4^ Moreover, Mexican children have high access to ultra-processed foods and limited access to safe areas, encouraging physical inactivity.^1^^,^^4^ This results in diets that are high in energy density, with elevated sugar and fat content, and low in micronutrient intake. The ENSANUT has reported that micronutrient deficiencies affect 56% of preschool-aged children, posing significant health risks and negatively impacting their physical and mental development.^5^ Furthermore, in combination with obesity, these deficiencies can contribute to the development of conditions such as nonalcoholic fatty liver disease,^6^ dyslipidemia, and cardiovascular diseases.^7^ This paradox exemplifies the double burden of malnutrition, where undernutrition (eg, micronutrient deficiencies) and overnutrition coexist within the same population.^8^

Although a few reviews have evaluated the association between obesity and micronutrient deficiencies,^9^^,^^10^ these tend to include only English-language studies, thus disregarding valuable information from other vulnerable low- and middle-income countries. Given the high prevalence of obesity and micronutrient deficiencies in Mexico, it is important to study the potential associations between these conditions within the Mexican context. This work is part of the “Childhood and Adolescent Obesity in Mexico: Evidence, Challenges, and Opportunities” (COMO) Project,^3^^,^^4^^,^^11–15^ which aims to synthesize and use data to comprehend the extent, nature, effects, and costs of childhood or adolescent obesity in Mexico. This study aims to systematically review the association between overweight or obesity and micronutrient deficiencies in the pediatric population of Mexico.

## METHODS

### Search Strategy

This work followed the Preferred Reporting Items for Systematic Reviews and Meta-Analyses (PRISMA) guidelines,^16^ and the protocol has been registered at the International Prospective Register of Systematic Reviews (PROSPERO registration no. CRD42019154132).^17^ The strategy for systematically reviewing existing evidence was based on the Population, Exposure, Comparison, Outcome, and Study Design (PECOS) framework described in Table 1.

**Table 1.** PECOS Criteria

	Inclusion criteria	Exclusion criteria
Population	Children and adolescents ≤18 y old of any sex, race, or ethnicity residing in Mexico	Individuals diagnosed with eating disorders, critical medical disorders (such as Down’s syndrome, NAFLD, cancer, CVD, HIV), or pregnant adolescents
Exposure	Overweight or obesity assessed using any anthropometrical measurement such as weight and height to estimate BMI, waist circumference, or fat percentage and classified according to national or international standards	Studies that do not clearly distinguish between normal weight, overweight, and obesity classifications (eg, those studies using BMI as a continuous variable without classifying the participants according to their BMI into a nutritional group)
Comparison	Any or none	N/A
Outcomes	Studies reporting micronutrient deficiencies measured through blood analysis, dietary evaluation, or physical deficiency symptoms	Studies evaluating anemia only use hemoglobin, as this condition is not specific to iron deficiency
Study design	Observational studies	Any other study design

Abbreviations: BMI, body mass index; CVD, cardiovascular disease; HIV, human immunodeficiency virus; N/A, not applicable; NAFLD, nonalcoholic fatty liver disease.

A sensitive search was developed using terms such as “micronutrients,” “vitamin,” “mineral,” “overweight,” “obesity,” “child,” “adolescent,” and “Mexico” connected through Boolean operators. (Full search strategy provided in Appendix S1.) A comprehensive and systematic search was performed using databases, including Medline, Embase, the Cochrane Library, Global Health Library, LILACS, CINAHL, CAB abstracts, ERIC, PsycINFO, ScienceDirect, Scopus, AGRICOLA, and SciELO Citation Index up to May 2024. Also, relevant material was searched using the Google Scholar search engine. When possible, searches were also conducted in Spanish to capture relevant references. Additionally, the COMO project database was used to locate relevant studies that may exist in Spanish or nonindexed journals.^11^ Conference abstracts were included if the required information was provided.

### Data Collection and Extraction

Titles and abstract screening and full-text review were performed by two reviewers (A.H.-G. and M.A.-M.). One reviewer extracted data (A.H.-G.) and a second reviewer (M.A.-M.) revised 100% of this extraction. A spreadsheet was designed using the PECOS framework to extract relevant data from the included studies. It comprised population characteristics (eg, sample size, gender, age), exposure metrics (eg, body mass index [BMI], waist circumference, fat percentage), outcomes (for each micronutrient: measurement method [blood analysis, dietary evaluation, or physical signs or symptoms of deficiency], criteria for deficiency, and overall result), and study design.

### Risk-of-Bias Analysis

The JBI (formerly the Joanna Briggs Institute) critical appraisal tool for cross-sectional studies was used to assess the quality of included studies.^18^ This tool appraises the methodological quality of studies and determines if the possibility of bias has been addressed throughout the study. The tool evaluates eight critical items: explicit inclusion and exclusion criteria, details about participants and setting, measurement of exposure methods, measurement of the condition, identification of confounding factors and strategies to deal with them, outcome measurement validity and reliability, and statistical analysis appropriateness. All studies were included regardless of their quality; however, this was considered during the synthesis process.

### Data Synthesis and Analysis

Data from the included studies were synthesized narratively, and crucial characteristics were tabulated. In addition, textual descriptions of studies and reported statistical analyses were recorded and tabulated. Reported outcomes of micronutrients and methods to measure the levels of such micronutrients are reported narratively in the Results section. As reported previously,^3^^,^^12^^,^^19^ BMI classification in children and adolescents may vary according to the included participants’ age or the references used to categorize BMI. Some studies use the categories “underweight,” “normal weight,” “at risk of overweight,” and “overweight,” while others classify BMI as “underweight,” “normal weight,” “overweight,” and “obesity.” For synthesis purposes, the categories “at risk of overweight” and “overweight” were unified in this review, and the categories “overweight” and “obesity” refer to children and adolescents in the two highest BMI categories, respectively, regardless of the anthropometric reference used in the studies.

### Statistical Analysis

Studies reporting deficiencies in vitamin D levels (measured through blood samples) were included in a random-effects meta-analysis. If studies reported raw data or odds ratios (ORs) on the likelihood of vitamin D deficiency based on BMI status, these were pooled into a primary analysis where higher BMI categories (overweight and/or obesity) were compared with lower BMIs (underweight and/or normal weight) data. The analysis was performed using the library “*meta*” with R statistical software (R Foundation for Statistical Computing, Vienna, Austria), and the main results are presented in forest plots.

## RESULTS

**Figure 1.**
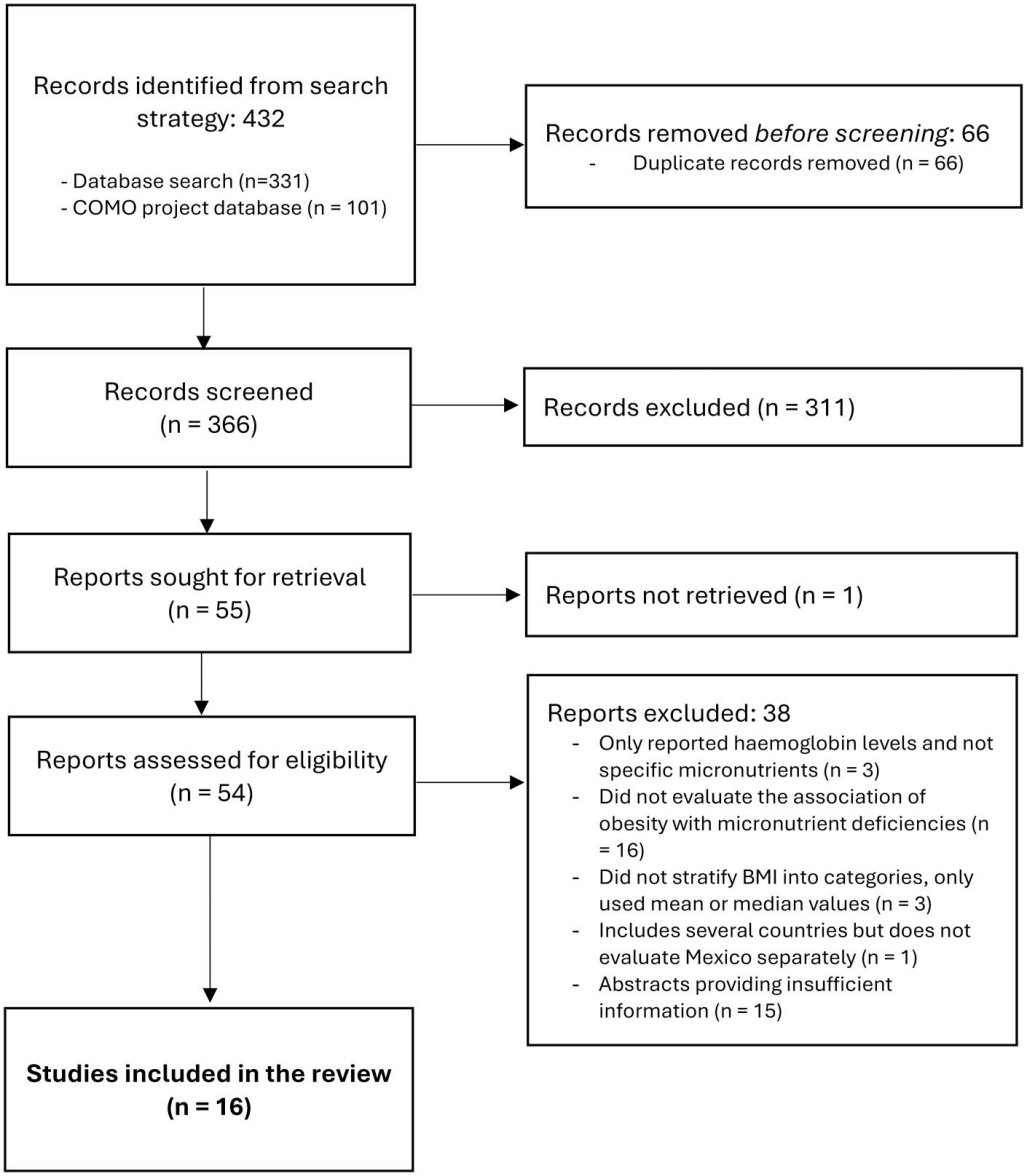
PRISMA Flowchart. Abbreviations: BMI, body mass index; COMO, Childhood and Adolescent Obesity in Mexico: Evidence, Challenges, and Opportunities; PRISMA, Preferred Reporting Items for Systematic Reviews and Meta-Analyses

**Figure 2.**
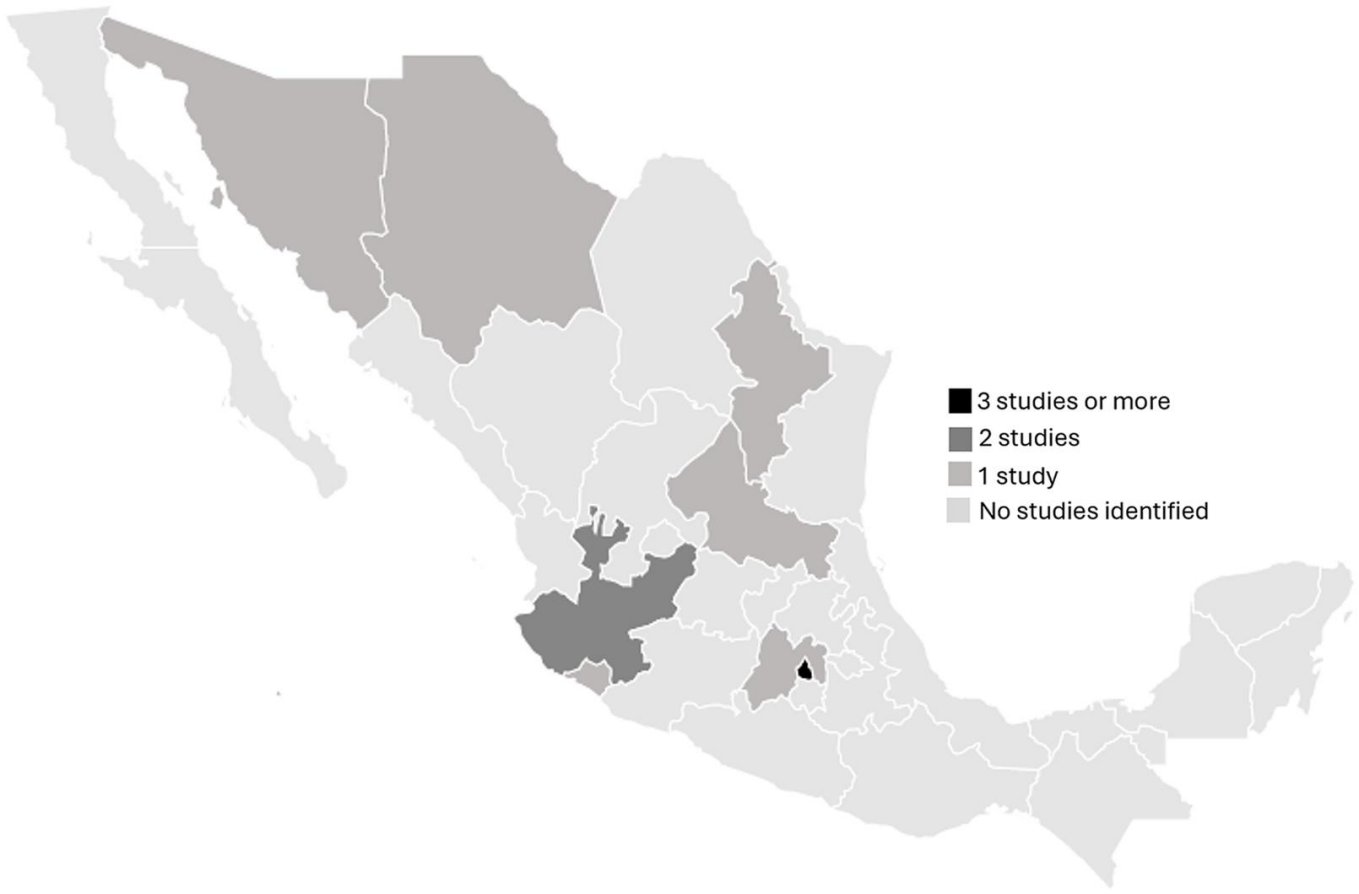
Map of the Evidence

A total of 366 unique references were identified from the literature search, of which 55 were retrieved for full-text screening, and 16 studies^5^^,^^20–34^ met the eligibility criteria and were included in this review (Figure 1). The study sample size varied from 44^33^ to 8716,^25^ including studies with nationally representative samples. From the included studies, 5 out of 16 included analyses from the ENSANUT^5^^,^^20^^,^^25^^,^^26^^,^^28^, and the rest included smaller-scale studies from Mexico City^21^^,^^22^^,^^30^ and other states, as shown in Figure 2. All of the studies included female and male participants, and no significant differences were observed between sexes. One study^31^ included only Indigenous children, and 1 abstract^29^ reported recruiting participants from rural areas (Table 2).

**Table 2.** General Characteristics of Studies

Study (year), study design	Setting	Population	OW/OB prevalence and reference used	Outcome	Measurement method
Cepeda-Lopez et al (2011),^20^ cross-sectional (ENN 1999)	National sample Home setting	*n* = 1174, 48.3% girls Mean age: NW: 8.23 (SD, 1.88); OW: 7.94 (SD, 1.97); OB: 8.34 (SD, 1.80)	OW: 18.1%; OB: 3.5% WHO	Iron, calcium, and vitamin C deficiency	Blood analysis (Hb, serum iron, TIBC, %TS) + dietary evaluation (validated 24HR)
Clark et al (2021),^21^ cross-sectional	Toluca, State of Mexico, and Mexico City Daycare centers, public and private schools, and a dental clinic (patients’ siblings)	*n* = 275, 45% girls Median age: PS: 4 y (3–5 y), SC: 8 y (7–9 y), adolescents: 13 y (12–14 y)	OW: 14.9%; OB: 12% WHO	Vitamin D deficiency	Blood analysis (serum 25-hydroxyvitamin D)
De la Cruz-Góngora et al (2021),^5^ cross-sectional (ENN 1999, ENSANUT 2006, ENSANUT 2018–19)	National sample Home setting	Age range: 1–4 y ENN 1999: *n* = 158; 43.6% females, 40.5% rural ENSANUT 2006: *n* = 1435; 46.9% females, 54% rural ENSANUT 2018–19, *n* = 1004; 47% females, 42% rural	ENN 1999: OW, 6.96% ENSANUT 2006: OW, 8.15% ENSANUT 2018–2019: OW, 7.17% WHO	Zinc deficiency	Blood analysis (serum zinc)
Denova-Gutiérrez et al (2019),^22^ cross-sectional	Mexico City Schools	*n* = 533, 45.8% females Mean age: 11.6 y (SD, 3.9 y)	OW: 22.1%; OB: 9% WHO	Vitamin D deficiency	Blood analysis (serum 25-hydroxyvitamin D)
Elizondo-Montemayor et al (2010),^23^ cross-sectional	Monterrey, Nuevo Leon 6 Public urban schools	*n* = 198, 49.5% females Mean age: participants with obesity: 8.99 y (SD, 1.997); participants without obesity 8.92 y (SD, 2.019)	OB: 50% WHO	Vitamin D deficiency	Blood analysis (serum 25-hydroxyvitamin D)
Ezzahra Housni et al (2022),^24^ cross-sectional	Chihuahua, Chihuahua 7 Public schools	*n* = 719; 50.9% females Mean age: females: 9.23 y (SD, 0.11); boys: 8.94 y (SD, 0.97)	OW: 16.83%; OB 10.99% WHO	Iron and vitamins A, C, and B-complex deficiency	Physical signs of deficiency for each micronutrient
Flores et al (2009),^25^ cross-sectional (ENSANUT 2006)	National sample Home setting	*n* = 8716, 50.6% females Mean age: 8.21 y (SD, 1.97) 29.4% rural	OW/OB: 25% Cole et al's^35^ sex- and age-specific BMI cutoff values (based on adult BMI classifications of OW/OB)	Zinc, iron, calcium, and vitamins A and C deficiency	Dietary evaluation (validated FFQ)
Flores et al. (2017),^26^ cross-sectional (ENSANUT 2012)	National sample Home setting	*n* = 2541 (PS: *n* = 1241; SC: *n* = 1300) Mean age: PS: 3 y (SE, 0.0); SC: 8.7 y (SE, 0.1)	PS: NW, 90.5%; OW, 9.5% SC: NW, 63.6%; OW, 19.9%; OB, 16.5% WHO	Vitamin D deficiency	Blood analysis (serum 25-hydroxyvitamin D) + dietary evaluation (validated FFQ)
Flores Ruelas et al (2020),^27^ cross-sectional	Colima, Colima 4 Public schools	*n* = 227, 48.5% females Median age: 9.27 y	OB: Children, 55.9%; adolescents, 22.9% WHO	Vitamin D deficiency	Blood analysis (serum 25-hydroxyvitamin D)
Flores et al (2021),^28^ cross-sectional (ENSANUT 2018–19)	National sample Home setting	*n* = 4691 (PS: *n* = 1209; SC: *n* = 3482); 50.6% females Mean age: PS: 2.8 y; SC: 8.2 y Rural: PS: 28.6%; SC: 27%	OW: PS, 5.4%; SC, 19.6% OB: SC, 17.9% WHO	Vitamin D deficiency	Blood analysis (serum 25-hydroxyvitamin D)
García et al (2011),^29^ (abstract), cross-sectional	Rural Mexico (no further data provided)	*n* = 199, gender NR Age range: 6–10 y	Not specified	Iron and vitamins A, C, and E deficiency	Blood analysis (not specified)
Martínez-Navarro et al (2023),^30^ cross-sectional	Mexico City 2 Public elementary schools and 2 private elementary schools	*n* = 210, 50% females Median age: 8 y (SD, 1.1)	OW/OB: 54.28% WHO	Zinc deficiency	Blood analysis (serum zinc)
Rodríguez Ramos al (2013),^31^ cross-sectional	Cuatlamayan, San Luis Potosi Public school Indigenous community	*n* = 84, 59.5% females Mean age: 13.7 y (SD, 1.3)	OW: 8.3% WHO	Zinc, iron, phosphorous, magnesium, calcium manganese, and B-complex deficiency	Dietary evaluation (validated FFQ)
Valdez López et al (2012),^32^ cross-sectional	Guadalajara, Jalisco Public high school	*n* = 307, 42.3% females Mean age: 13 y (SD, 2.67)	OW: 8.1%; OB: 19.8% NR	Iron, calcium, zinc, and vitamins A, C, B_2_, B_6_, B_9_, and B_12_ deficiency	Dietary evaluation (24HR)
Valle-Leal et al (2017),^33^ cross-sectional	Obregon, Sonora Public hospital	*n* = 44, 45% females Mean age: 9.6 y (SD, 1.72)	OW: 18%, OB: 82% WHO	Vitamin D deficiency	Blood analysis [serum 1,25(OH)_2_ vitamin D]
Vargas-Hernández et al (2013),^34^ cross-sectional	Guadalajara, Jalisco Public school	*n* = 125, 60.8% females Mean age: 13.5 y (SD, 0.9)	OW: 37.6% CDC	Calcium deficiency	Dietary evaluation (24HR, validated FFQ)

Abbreviations: BMI, body mass index; CDC, Centers for Disease Control and Prevention; ENN, National Nutrition Survey; ENSANUT, National Health and Nutrition Survey; FFQ, food-frequency questionnaire; Hb, hemoglobin; NR, not registered; NW, normal weight; OB, obesity; OW, overweight; PS, preschoolers; SC, schoolchildren; TIBC, total iron-binding capacity; TS, transferrin saturation; WHO, World Health Organization; 24HR, 24-hour recall.

Table 2 shows an overview of the micronutrients studied across the included studies. Table 3 summarizes the methods to measure micronutrient deficiency and the overall association between the different micronutrient deficiencies and overweight or obesity. The following sections are organized based on the evidence found for each micronutrient, divided into minerals and vitamins.

**Table 3.** Exposure and Outcomes Association in the Included Studies

Micronutrient and study (year)	Outcome measurement method		Summary of outcomes
***Zinc***			
De la Cruz-Góngora et al (2021)^5^	Blood analysis	Serum zinc	OW was significantly associated with serum zinc deficiency (OR, 2.18; 95% CI, 1.11–4.26; *P* < .023).
Martínez-Navarro et al (2023)^30^	Blood analysis	Serum zinc	Children with NW and OW/OB presented deficiency, but children with OW/OB had significantly lower serum levels (*P* = .033). A significant association was also found between a larger WC and deficient zinc levels (*P* = .038).
Rodríguez Ramos et al (2013)^31^	Dietary evaluation	Validated FFQ	Significantly lower zinc intake in children with OW (*P* = .048), with 100% of them consuming deficient zinc amounts (*P* = .008).
Flores et al (2009)^25^	Dietary evaluation	Validated FFQ	Tendency for deficient intake in children with OW, but no statistical significance was found.
Valdez López et al (2012)^32^	Dietary evaluation	24HR	Important deficiencies across all BMI groups, particularly in children with NW. Statistical analysis was not performed.
***Iron***			
Cepeda-López et al (2011)^20^	Blood analysis + dietary evaluation	Hb, serum iron, TIBC, %TS + validated 24HR	OB was a significant predictor of iron deficiency when evaluated through a blood sample (OR, 2.96; 95% CI, 1.34–11.67; *P* < .05). No differences in dietary intake were found between BMI groups.
García et al (2011)	Blood analysis	Not specified	Did not find important deficiencies in any BMI category.
Rodríguez Ramos et al (2013)^31^	Dietary evaluation	Validated FFQ	Important deficiencies were observed across all BMI groups, but the differences between groups were not significant.
Valdez López et al (2012)^32^	Dietary evaluation	24HR	Important deficiencies across all BMI groups. No statistical analysis was provided.
Flores et al (2009)^25^	Dietary evaluation	Validated FFQ	Relevant deficiencies were not found in any groups. Potential underestimation due to a higher iron bioavailability assumption.
Ezzahra Housni et al (2022)^24^	Physical signs	Paleness of skin, tongue conjunctiva, and koilonychia	Children with OW/OB were more likely to have iron deficiency (OR, 164; 95% CI, 41.66–647.11; *P* < .001).
***Calcium***			
Vargas-Hernandez et al (2013)^34^	Dietary evaluation	24HR + validated FFQ	A higher percentage of adolescents with OW/OB had a deficient intake (<900 mg/d) compared with those with an elevated intake (>1300 mg/d) (*P* < .05). A higher percentage of adolescents with NW had an elevated intake (>1300 mg/d) (*P* < .05).
Cepeda-López et al (2011)^20^	Dietary evaluation	Validated 24HR	Calcium intakes were deficient in all children, and differences were not significant between BMI groups.
Rodríguez et al (2013)^31^	Dietary evaluation	Validated FFQ	A high percentage of deficiency was seen in all BMI groups, and although 100% of children with OW presented deficient intake, no statistical difference was found.
Flores et al (2009)^25^	Dietary evaluation	Validated FFQ	Important deficiencies across all BMI categories, with no statistical difference between groups.
Valdez López et al (2012)^32^	Dietary evaluation	24HR	Important deficiencies across all BMI groups, especially in UW females (82.7%) and in males with OW/OB (85.1%). No statistical analysis was provided.
***Phosphorous***			
Rodríguez Ramos et al (2013)^31^	Dietary evaluation	Validated FFQ	A high percentage of deficiencies was observed across all BMI groups, with no significant differences between groups.
Magnesium			
Rodríguez Ramos et al (2013)^31^	Dietary evaluation	Validated FFQ	A high percentage of deficiencies was observed across all BMI groups, with no significant differences between the groups. The mean daily intake was reported to be the lowest in children with OW (*P* = .046).
Manganese			
Rodríguez Ramos et al (2013)^31^	Dietary evaluation	Validated FFQ	The majority of children with OW exceeded recommended manganese intake levels, with no significant differences between BMI categories. The mean daily intake was reported to be the lowest in OW children (*P* = .005).
***Vitamin D***			
Flores et al (2017)^26^	Blood analysis + dietary evaluation	Serum 25-hydroxyvitamin D + validated FFQ	The prevalence of deficiency in school-age children was markedly higher in children with OW (47.7%) compared with those with a normal BMI (29.5%) (*P* < .05). Mean vitamin D intake was higher among children with OW (*P* < .05).
Elizondo-Montemayor et al (2010)^23^	Blood analysis	Serum 25-hydroxyvitamin D	Individuals with obesity were significantly less likely to have optimal levels (*P* < .001) and significantly more likely to have deficient levels compared with nonobese individuals (*P* = .021).
Denova-Gutiérrez et al (2019)^22^	Blood analysis	Serum 25-hydroxyvitamin D	A significant association between obesity and lower vitamin D levels was found. A significant proportion of individuals with OW and OB presented deficient levels compared with insufficient levels (*P* < .01).
Flores et al (2021)^28^	Blood analysis	Serum 25-hydroxyvitamin D	Schoolchildren with OW/OB were significantly more likely to have insufficient levels than optimal levels (*P* < .05). Individuals with NW were more likely to have sufficient levels than deficient levels (*P* < .05).
Flores Ruelas et al (2020)^27^	Blood analysis	Serum 25-hydroxyvitamin D	Children with WC <90th percentile showed a prevalence of hypovitaminosis in 28.30% of cases, while 71.70% had sufficient levels. The statistical significance of these findings was not described.
Valle-Leal et al (2017)^33^	Blood analysis	Serum 1,25(OH)_2_ vitamin D	No association was found between increased adiposity and low levels of 1,25(OH)_2_ vitamin D.
Clark et al (2021)^21^	Blood analysis	Serum 25-hydroxyvitamin D	No significant difference in vitamin D levels was found between children with a BMI *z*-score <1 and those with a BMI *z*-score ≥1 across all age groups.
Vitamin E			
García et al (2011)^29^	Blood analysis	Not specified	The percentage of children with OW/OB and vitamin E deficiency was not significantly different from their peers with NW.
***Vitamin A***			
García et al (2011)^29^	Blood analysis	Not specified	Low deficiency prevalence across all BMI groups (10% NW, 4.1% OW/OB), with no significant differences between them.
Ezzahra Housni et al (2022)^24^	Physical signs	Conjunctival xerosis, bitot spots, brittle skin, follicular hyperkeratosis	OW/OB could be associated with lower odds of vitamin A deficiency (OR = 0.26), but this was not statistically significant.
Flores et al (2009)^25^	Dietary evaluation	Validated FFQ	The prevalence of dietary inadequacy was 46.6% for those with normal BMI, 43.3% for OW, and 43.8% for OB. There were no statistical differences between groups.
Valdez López et al (2012)^32^	Dietary evaluation	24HR	Important deficiencies across all BMI groups, with the highest prevalences in males with NW (72.6%) and OW/OB (72.3%), but no statistical analysis was provided.
***Vitamin C***			
García et al (2011)^29^	Blood analysis	Not specified	Deficiency was not highly prevalent in any of the groups, and differences in between groups were not significant.
Cepeda-López et al (2011)^20^	Dietary evaluation	Validated 24HR	Significantly higher intake in the OW/OB groups compared with the NW category (*P* = .001).
Flores et al (2009)^25^	Dietary evaluation	Validated FFQ	Deficiency was not highly prevalent in any of the groups, and differences in between groups were not significant.
Valdez López et al (2012)^32^	Dietary evaluation	24HR	In the undernutrition (71.4%) and OW/OB (74.4%) categories, deficiency was more frequently observed in males. Statistical analysis was not reported.
Ezzahra Housni et al (2022)^24^	Physical signs	Swollen or bleeding gums	The likelihood (OR) that children with OW/OB had a vitamin C deficiency was not significant.
***B-complex vitamins***			
Rodríguez Ramos et al (2013)^31^	Dietary evaluation	Validated FFQ	Significant lower intake of vitamin B_6_ among adolescents with OW (*P* = .022), with 100% of them consuming deficient amounts of the micronutrient. Most adolescents reached the recommended folate intake. Adolescents with OW had the lowest intake among those meeting sufficient levels, but the differences were not statistically significant.
Valdez López et al (2012)^32^	Dietary evaluation	24HR	Important folate deficiencies existed in all categories, particularly in females with OW/OB (85.3%) and UW (82.7%) and males with NW (76.8%). Pyridoxine deficiency was notable in all groups, especially in females with OW/OB (56%) and males with OW/OB (46.8%). Cobalamin deficiency was present particularly in females with NW (40%) and males with OW/OB (34%). Relatively low riboflavin deficiency was documented, with higher percentages in NW females (30%) and males with OW/OB (25.5%). Statistical analysis was not reported for any of these results.
Ezzahra Housni et al (2022)^24^	Physical signs	Nasolabial seborrhea, glossitis, magenta tongue, papillary atrophy, angular stomatitis, cheilosis	Children with OW/OB were significantly more likely to have B-complex vitamin (B_2_, B_3_, B_6_) deficiency (OR, 23.48; 95%CI, 6.49–84.91; *P* < .001).

Abbreviations: BMI, body mass index; FFQ, food-frequency questionnaire; Hb, hemoglobin; NW, normal weight; OB, obesity; OR, odds ratio; OW, overweight; TIBC, total iron binding capacity; TS, transferrin saturation; UW, underweight; WC, waist circumference; 24HR, 24-hour recall.

### Minerals

#### Zinc

Five studies measured the association between overweight or obesity and zinc deficiency.^5^^,^^25^^,^^30–32^ Two studies used serum zinc values and applied international cutoffs to determine the deficiency^5^^,^^30^; the other studies evaluated deficiency through validated dietary assessment methods.^25^^,^^31^^,^^32^ Three of these studies reported that children who had overweight or obesity also had a statistically nonsignificant higher prevalence of zinc deficiency.^5^^,^^30^^,^^31^ Additionally, Flores et al^25^ reported a tendency for deficient intake in children with overweight, but no statistical significance was found. Moreover, Rodríguez Ramos et al^31^ found a significantly lower zinc intake in children with overweight (*P* = .048); however, most participants in the study were reported to be consuming inadequate amounts of zinc (*P* = .008).

#### Iron

Six studies investigated iron status in children with overweight or obesity.^20^^,^^24^^,^^25^^,^^29^^,^^31^^,^^32^ Results were highly mixed, as two studies identified overweight or obesity as a significant predictor for iron deficiency.^20^^,^^24^ However, the methods for measuring iron levels varied across studies. One of these studies did report that obesity was a significant predictor of iron deficiency when evaluated through biochemical indicators (OR, 2.96; 95% CI, 1.34–11.67), but no differences in dietary intake were found between BMI groups.^20^ Instead, they found that C-reactive protein (CRP) was positively associated with BMI and negatively with iron status.^20^ Two other studies found a high prevalence of iron deficiency across all BMI groups, with no significant difference for children with overweight or obesity,^31^^,^^32^ but 2 additional studies contradicted these results and concluded that there were no significant iron deficiencies in any group.^25^^,^^29^

#### Calcium

Five studies evaluated the association between overweight or obesity and calcium deficiency.^20^^,^^25^^,^^31^^,^^32^^,^^34^ Only one study reported a significantly higher number of adolescents with overweight or obesity in the deficient-calcium-intake category than in other intake categories (*P* < .05).^34^ The rest of the studies reported important deficiencies across all BMI categories, with no significant difference reported across groups.

#### Phosphorus, magnesium, and manganese

Only one study evaluated the association between overweight or obesity and phosphorus, magnesium, and manganese deficiency.^31^ A high percentage of children with phosphorus and magnesium deficiencies were observed across all BMI groups, with no significant differences. All children exceeded the recommended intake levels for manganese. The mean daily intake of these minerals was reported to be the lowest in children who were overweight; however, this was only significant for magnesium (*P* = .046) and manganese (*P* = .005).

### Vitamins

#### Vitamin D

**Figure 3.**
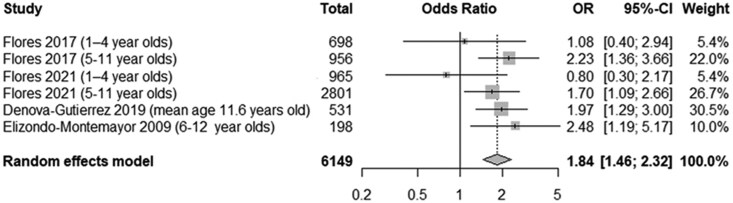
Forest Plot of Vitamin D Deficiency (All Participants). Abbreviation: OR, odds ratio

**Figure 4.**
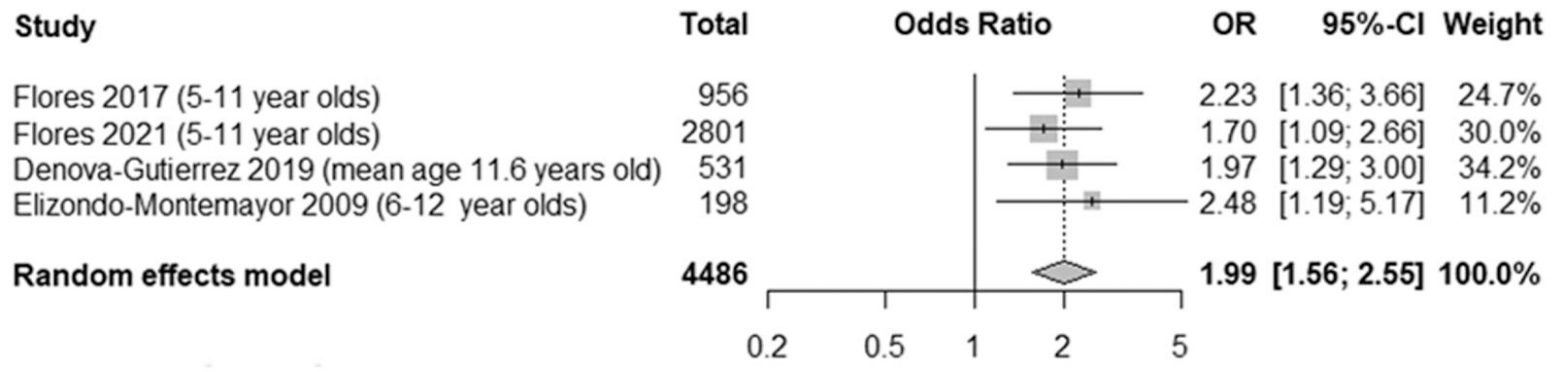
Forest Plot of Vitamin D Deficiency (School-Aged Children). Abbreviation: OR, odds ratio

Seven studies measured the association between overweight or obesity and vitamin D deficiency.^21–23^^,^^26–28^^,^^33^ All of them included children with normal weight, except for two studies^22^^,^^33^ that included participants with obesity only. Four studies found a significant association between higher BMI and deficient vitamin D levels (*P* < .05).^22^^,^^23^^,^^26^^,^^28^ Two of these studies, which used data from ENSANUT, stated that this finding was only significant for school-aged children and not for preschoolers.^26^^,^^28^ Nevertheless, one of these studies additionally reported a higher vitamin D intake among children of all ages with overweight.^26^ Another study found that children with a waist circumference below the 90th percentile showed a lower prevalence of hypovitaminosis,^27^ but the statistical significance of these findings was not reported. Two other studies could not prove the association of vitamin D deficiency in children with overweight or obesity.^21^^,^^33^ Among the 7 studies that analyzed the relationship of overweight or obesity with vitamin D deficiency, four studies^22^^,^^23^^,^^26^^,^^28^ that used similar methods of measuring vitamin D were included in a random-effects meta-analysis (Figure 3). Overall, children with overweight and/or obesity were found to have a significantly higher likelihood of vitamin D deficiency (OR, 1.84; 95% CI, 1.46–2.32). Additionally, we found that this likelihood was higher and remained significant when the analysis was conducted only in school-aged children (OR, 1.99; 95% CI, 1.56–2.55) (Figure 4).

#### Vitamins A and E

Four studies evaluated the association between overweight or obesity and vitamin A deficiency.^24^^,^^25^^,^^29^^,^^32^ The results of these studies were highly mixed, and none found a significant difference. One study reported a low prevalence of deficiency across all BMI groups.^29^ Another study found that overweight or obesity could be associated with a lower risk of vitamin A deficiency.^24^ One study reported notable vitamin A deficiencies across all BMI groups.^25^ One final study found important deficiencies across all BMI groups, with the highest prevalences in males with normal weight, overweight, or obesity.^32^ Only one study evaluated vitamin E deficiency in children with overweight or obesity and found that their deficiency was not significantly different from that of their peers with normal weight.^29^

#### Vitamin C

Five studies evaluated the association of overweight or obesity with vitamin C deficiency.^20^^,^^24^^,^^25^^,^^29^^,^^32^ Three of these studies agreed that children with overweight or obesity did not have a higher prevalence of deficiency.^20^^,^^25^^,^^29^ For instance, Cepeda-López et al^20^ found a significantly higher vitamin C intake in the overweight or obesity groups compared with children in the normal-weight category (*P* < .05). Another study confirmed that the association between overweight or obesity and vitamin C deficiency was not significant.^24^ In contrast, one study reported that vitamin C deficiency was highly prevalent, especially in males, in both the undernutrition and overweight or obesity categories, but no statistical significance was reported.^32^

#### B-Complex Vitamins

Three studies evaluated the association of overweight or obesity with vitamin B deficiencies.^24^^,^^31^^,^^32^ One study found that children with overweight or obesity had a higher risk of B-complex vitamin deficiency (OR, 23.48; 95% CI, 6.49–84.91).^24^ Another study also reported significantly lower pyridoxine intake among adolescents with overweight (*P* = .022), with all of the participants consuming deficient amounts of this micronutrient.^31^ However, most adolescents in this study met the recommended daily folate intake levels. A third study found important B-complex vitamin deficiencies across all categories, especially folate and pyridoxine. Cobalamin and riboflavin deficiencies were also noted, but to a lesser extent; however, no statistical significance was reported for these findings.^32^

### Quality of Included Studies

Out of 16 studies, 11 were considered high quality,^5^^,^^20^^,^^22–26^^,^^28^^,^^30^^,^^34^ one study was of unclear quality,^21^ and four studies were low quality.^29^^,^^31–33^ All studies, except for two,^29^^,^^32^ clearly described the inclusion criteria, study participants, and setting. The most common reason for studies being classified as unclear or low quality was the failure to identify or address confounding factors.^31–33^ Most studies used reliable methods to evaluate the exposure (ie, overweight or obesity) and the outcomes (ie, micronutrient deficiencies) (Table 4).

**Table 4.** Risk-of-Bias Assessment

Study (year)	Were the criteria for inclusion in the sample clearly defined?	Were the study participants and the setting described in detail?	Was the exposure measured in a valid and reliable way?	Were objective, standard criteria used for measurement of the condition?	Were confounding factors identified?	Were strategies to deal with confounding factors stated?	Were the outcomes measured in a valid and reliable way?	Was appropriate statistical analysis used?
Cepeda-López et al (2011)^20^	✔	✔	✔	✔	✔	✔	✔	✔
Clark et al (2021)^21^	✔	✔	✔	✔	**?**	**?**	✔	✔
De la Cruz-Góngora et al (2021)^5^	✔	✔	✔	✔	✔	✔	✔	✔
Denova-Gutiérrez et al (2019)^22^	✔	✔	✔	✔	✔	✔	✔	✔
Elizondo-Montemayor et al (2010)^23^	✔	✔	✔	✔	✔	✔	✔	✔
Ezzahra Housni et al (2022)^24^	✔	✔	✔	✔	✔	✔	✔	✔
Flores et al (2009)^25^	✔	✔	✔	✔	✔	✔	✔	✔
Flores et al (2017)^26^	✔	✔	✔	✔	✔	✔	✔	✔
Flores Ruelas et al (2020)^27^	✔	✔	✔	✔	✔	✔	✔	✔
Flores et al (2021)^28^	✔	✔	✔	✔	✔	✔	✔	✔
García et al (2011)^29^ (abstract)	✗	✗	✔	**?**	✔	✔	**?**	✔
Martínez-Navarro et al (2023)^30^	✔	✔	✔	✔	✔	✔	✔	✔
Rodríguez Ramos et al (2013)^31^	✔	✔	✔	✔	✗	✗	✔	✔
Vargas-Hernandez et al (2013)^34^	✔	✔	✔	✔	✔	✔	✔	✔
Valle-Leal et al (2017)^33^	✔	✔	✔	✔	✗	✗	✗	✔
Valdez López et al (2012)^32^	✗	✗	✗	✗	**?**	**?**	**?**	✗

## DISCUSSION

This systematic review evaluated the association between overweight or obesity and micronutrient deficiencies in Mexican children and adolescents. A total of 16 studies met the inclusion criteria, of which the majority (68%) were of high quality. Results were highly mixed depending on the micronutrient studied or the methodology used for measuring micronutrient levels (eg, blood samples, dietary intake estimation, or signs or physical symptoms of deficiency). This review found that Mexican children with overweight or obesity are more likely to have vitamin D deficiency, especially if they are school-aged children. Moreover, evidence also suggests that children with overweight and obesity are also prone to zinc, iron, and vitamin B_6_ deficiencies. Importantly, deficiencies in calcium, phosphorus, magnesium, and vitamin E were observed, but these did not differ significantly across BMI categories. Additionally, a low deficiency prevalence was seen for manganese and vitamins A and C, with no significant differences across BMI categories. Finally, limited evidence was found for certain micronutrients (eg, phosphorus, magnesium, and vitamin E), indicating that further research is necessary to determine any possible association.

Micronutrient deficiencies during childhood can profoundly affect the developmental process, as these are essential for growth and development. For instance, zinc has crucial roles in various metabolic pathways and immune and cognitive functions, and its deficiency contributes to impaired growth.^36^ Calcium, phosphorus, and magnesium are also essential for bone development, and their deficiency has been linked to a higher risk of developing rickets,^37^ especially when vitamin D is also deficient.^38^ Beyond bone health, calcium has also been studied for its potential anti-obesity effects. A meta-analysis suggested that sufficient calcium intake may help prevent obesity, with stronger effects observed in lean individuals compared with those who have overweight or obesity.^39^ However, additional high-quality observational and longitudinal studies are necessary to clarify whether calcium deficiency plays a causal role in the development of obesity or is a consequence of it.

Vitamins A, E, and C are involved in inflammatory processes, protecting against oxidative stress,^40–42^ which has been reported to be elevated in individuals with obesity and is a potential risk factor for related complications.^42^ A cross-sectional study in Mexican children found that low levels of vitamins A and E and zinc in those with overweight or obesity were associated with adverse lipid profiles, increased inflammation, and insulin resistance.^43^ Additionally, vitamin C is important because it enhances iron absorption and helps reduce the risk of anemia. Vitamin C improves non-heme iron absorption by reducing ferric iron (Fe^3+^) to ferrous iron (Fe^2+^), which is more soluble and readily absorbed by the intestine.^44^ This is particularly relevant in Mexico, where high maize consumption, rich in phytates, can further hinder iron absorption.^44^^,^^45^

Other systematic reviews evaluating children from different nationalities have found results similar to those in the current review, concluding that overweight or obesity is associated with certain micronutrient deficiencies. For example, Tan et al^10^ concluded that overnutrition in children was associated with an increased risk of iron deficiency but not zinc or vitamin A deficiency. Zhao et al^46^ also confirmed a strong correlation between obesity and the risk of developing iron deficiency in individuals under 18 years. The difference in zinc results could have been related to the higher phytate levels in the Mexican diet, which contribute to zinc and iron deficiency.^45^^,^^47^

When meta-analyzing data, we found that children, especially school-aged children, with overweight or obesity were more likely to have vitamin D deficiency. Vitamin D in individuals with obesity has been extensively studied, and our findings align with previous research.^9^^,^^48–51^ A few theories describe the phenomenon of vitamin D deficiency and obesity. Some authors suggest that deficiency might be due to reduced sun exposure, as individuals with obesity may engage less in outdoor activities or wear more clothing to cover their bodies.^52^ Limited sunlight exposure may also result from increased screen time, as children spend long hours indoors using electronic devices such as tablets and smartphones.^53^ Additionally, it is also important to consider that skin pigmentation influences vitamin D synthesis, and darker skin requires more sun exposure to produce adequate levels of vitamin D, making certain children more susceptible to deficiency under the same environmental conditions.^54^^,^^55^ One of the studies included in this review^23^ analyzed children’s skin types and found that most of them had skin phototype IV, which means they require longer periods of sun exposure to synthesize sufficient vitamin D. However, the most widely accepted pathophysiological explanation is that, as a fat-soluble vitamin, vitamin D becomes sequestered in adipose tissue, reducing its bioavailability in the circulation.^56^ Supporting this theory, a study by Wortsman et al^56^ showed that, after exposing participants with normal weight and obesity to ultraviolet-B radiation, the increase in serum vitamin D was 57% lower in those with obesity.^56^ It was concluded that the higher amount of subcutaneous fat in individuals with obesity may sequester vitamin D, leading to lower serum levels. This deficiency can have significant consequences, particularly in growing children. Vitamin D is crucial in calcium and phosphorus metabolism, essential for bone development and mineralization.^57^ Inadequate levels can lead to conditions such as rickets, characterized by bone deformities and impaired growth.^58–60^ Beyond skeletal effects, vitamin D deficiency has also been associated with an increased risk of insulin resistance^61^^,^^62^ and immune dysregulation.^60^^,^^63^^,^^64^ In children with obesity, these risks may be compounded, potentially exacerbating inflammatory processes and contributing to the early onset of chronic diseases.

With regard to other micronutrients, several cross-sectional studies from other countries evaluated vitamin A deficiency in the pediatric population and, contrary to this study’s findings, they reported lower levels of carotenoids and/or retinoids in children with obesity compared with normal-weight children.^65–68^ This discrepancy may be attributed to variations in the methodologies of the studies included in this review. Notably, the only study that measured vitamin A in blood did not specify which component was measured,^29^ and the remaining studies used less reliable evaluation methods for confirming vitamin A deficiency.^24^^,^^25^^,^^32^ Not many other studies have evaluated the association between pediatric obesity and vitamin C deficiency. Some nationally representative surveys from different countries have concluded an elevated prevalence of vitamin C deficiency in adults with obesity.^69^^,^^70^ However, most studies found in this review suggest high vitamin C levels among Mexican children with obesity, which may be linked to high consumption of sugary drinks in the country. The ENSANUT revealed that sugary drinks were the most consumed item among the non-recommended foods, with 82.6% of preschoolers, 93.6% of school-aged children, and 90.3% of adolescents consuming them.^71^ Additionally, the Federal Agency of Consumer Affairs (PROFECO) confirmed that these drinks provide up to 75 mg of vitamin C per serving, exceeding the recommended daily intake.^72^ Although this review did not identify a significant deficiency in vitamin C, it is crucial that future research continues to assess serum levels of this micronutrient in the pediatric population with obesity. In the present work, most included studies assessed vitamin C status based on dietary intake rather than biochemical markers, although previous literature has identified a potential risk of deficiency due to multiple factors, including reduced dietary intake, volumetric dilution related to increased body size, and elevated basal metabolic rate, which increases physiological demand for vitamin C.^73^

Not many other studies have evaluated the association between pediatric obesity and calcium, phosphorus, and magnesium deficiencies. Additionally, more research is needed to assess B-complex vitamin deficiency in Mexican children with obesity, as the current evidence is not conclusive. Cross-sectional studies from other countries have found a negative association between BMI and deficiencies in folate and cobalamin.^74–76^ Although the results from the included studies suggested a vitamin B_6_ deficiency in our population, comparing these findings with those of other studies was not possible, as the prevalence of vitamin B_6_ deficiency is unknown and is not commonly screened for.^77^

It is important to acknowledge some limitations of this study, particularly the considerable heterogeneity among the included studies. For instance, different methods were used to measure micronutrient deficiencies, and the lack of uniformity in methodologies and analyses hindered comparisons. As a result, this variability made a quantitative analysis impossible for some of the micronutrients analyzed in this review. Additionally, there was heterogeneity in the references used to classify the nutritional status of children (eg, World Health Organization, Centers for Disease Control and Prevention, or International Obesity Task Force). Research demonstrates substantial variations in the nutritional status classification of Mexican children, based on the reference used.^78^ While we consolidated certain categories for analysis purposes, it is crucial to recognize the methodological differences (such as the populations studied) in determining cutoff points and growth standards of the different references used. These differences may lead to variations in classifying overweight and obesity, affecting some of the results presented in this review. Moreover, all of the studies included in this review used a cross-sectional design, and due to the lack of longitudinal data, causality was not assessed. Also, the association between deficiencies and obesity might be bidirectional, and some nutritional deficiencies may contribute to weight gain. Similar to obesity and mental health, longitudinal data would be needed to better study such bidirectionality, which, unfortunately, is nonexistent within the Mexican context when examining obesity and the association with micronutrient deficiencies.^15^

Strengths of this work include being the first systematic review conducted to evaluate the association of obesity and micronutrient deficiencies in Mexican children and adolescents. Furthermore, a comprehensive search strategy was performed in English and Spanish, which was useful for extracting relevant publications for the country. Additionally, the COMO database^11^ provided access to relevant studies in the gray literature.

This work provides insight into the nutritional burden of childhood obesity in the Mexican context. Current evidence suggests that Mexican children with overweight or obesity are significantly more likely to have certain micronutrient deficiencies, such as vitamin D. These findings underscore the double burden of malnutrition in Mexican children, where excess body weight coexists with critical micronutrient deficiencies, increasing the risk of metabolic disorders, impaired development, and noncommunicable diseases.^79^ However, more evidence is needed to evaluate deficiencies using longitudinal analysis, biochemical analysis, and dietary assessments to determine their causality and measure the potential impact of these deficiencies on the pediatric population with obesity. The impact of these micronutrient deficiencies may be less frequently diagnosed in children with obesity, and the short- and long-term effects have not yet been studied. Understanding the potential micronutrient deficiencies in this population encourages healthcare professionals to consider them within nutritional intervention standards to prevent future complications related to growth, development, and metabolic processes, as well as to tackle this issue more comprehensively.

## Supplementary Material

nuaf148_Supplementary_Data

## Data Availability

The data and studies that support the findings of this systematic review and meta-analysis are openly available online, and then be revised in the reference list.

## References

[1] Shamah-Levy T , Gaona-Pineda EB, Cuevas-Nasu L, et al Prevalencias de sobrepeso y obesidad en población escolar y adolescente de México. ENSANUT Continua 2020-2022. Salud Públ México. 2023;65:s218-s224. 10.21149/14762 38060970

[2] Aceves-Martins M , Llauradó E, Tarro L, Solà R, Giralt M. Obesity-promoting factors in Mexican children and adolescents: challenges and opportunities. Glob Health Action. 2016;9:29625. 10.3402/gha.v9.29625 26787421 PMC4718931

[3] Aceves-Martins M , Gutierrez-Gómez YY, Moreno-García CF. Socioeconomic determinants of overweight and obesity among Mexican children and adolescents: systematic review and meta-analysis. Obes Rev. 2025;26:e13926. 10.1111/obr.13926 40210200 PMC12246893

[4] Aceves-Martins M , López-Cruz L, García-Botello M, Godina-Flores NL, Gutierrez-Gómez YY, Moreno-García CF. Cultural factors related to childhood and adolescent obesity in Mexico: a systematic review of qualitative studies. Obes Rev. 2022;23:e13461. 10.1111/obr.13461 35587773 PMC9541705

[5] De la Cruz-Góngora V , García-Guerra A, Shamah-Levy T, Villalpando S, Valdez-Echeverría R, Mejía-Rodríguez F. Estado de micronutrimentos en niños, niñas y mujeres mexicanas: análisis de la ENSANUT Continua 2022. Salud Públ México. 2023; 65:s231-s237. 10.21149/14781 38060968

[6] Yasutake K , Kohjima M, Kotoh K, Nakashima M, Nakamuta M, Enjoji M. Dietary habits and behaviors associated with nonalcoholic fatty liver disease. World J Gastroenterol. 2014;20:1756-1767. 10.3748/wjg.v20.i7.1756 24587653 PMC3930974

[7] Amorim De Farias Leal A , Camêlo Palmeira Á, Menezes Almeida De Castro G, Oliveira Da Silva Simões M, Teixeira Ramos A, Medeiros CCM. Homocysteine: cardiovascular risk factor in children and adolescents? Rev Assoc Méd Brasil. 2013;59:622-628. 10.1016/j.ramb.2013.05.004 24182942

[8] World Health Organization. The double burden of malnutrition: policy brief. 2017. May 17, 2017. Accessed October 8, 2024. https://www.who.int/publications/i/item/WHO-NMH-NHD-17.3

[9] Pereira‐Santos M , Costa P, Assis A, Santos C, Santos D. Obesity and vitamin D deficiency: a systematic review and meta‐analysis. Obes Rev. 2015;16:341-349. 25688659 10.1111/obr.12239

[10] Tan X , Tan PY, Gong YY, Moore JB. Overnutrition is a risk factor for iron, but not for zinc or vitamin A deficiency in children and young people: a systematic review and meta-analysis. BMJ Glob Health. 2024;9:e015135. 10.1136/bmjgh-2024-015135 PMC11015307 38599666

[11] Aceves-Martins M. The “Childhood and Adolescent Obesity in Mexico: Evidence, Challenges, and Opportunities” (COMO) Project. 2024. Accessed February 2, 2025. https://www.comoprojectmx.com/

[12] Aceves-Martins M , Godina-Flores NL, Gutierrez-Gómez YY, et al Obesity and oral health in Mexican children and adolescents: systematic review and meta-analysis. Nutr Rev. 2022;80:1694-1710. 10.1093/nutrit/nuab088 34664672 PMC9086795

[13] Aceves-Martins M , López-Cruz L, García-Botello M, Gutierrez-Gómez YY, Moreno-García CF. Interventions to prevent obesity in Mexican children and adolescents: systematic review. Prev Sci. 2022;23:563-586. 10.1007/s11121-021-01316-6 34725762 PMC9072495

[14] Aceves-Martins M , López-Cruz L, García-Botello M, Gutierrez-Gómez YY, Moreno-García CF. Interventions to treat obesity in Mexican children and adolescents: systematic review and meta-analysis. Nutr Rev. 2022;80:544-560. 10.1093/nutrit/nuab041 34339511 PMC8829677

[15] Godina-Flores NL , Gutierrez-Gómez YY, García-Botello M, López-Cruz L, Moreno-García CF, Aceves-Martins M. Obesity and its association with mental health among Mexican children and adolescents: systematic review. Nutr Rev. 2023;81:658-669. 36164834 10.1093/nutrit/nuac083 PMC10170326

[16] Page MJ , McKenzie JE, Bossuyt PM, et al The PRISMA 2020 statement: an updated guideline for reporting systematic reviews. BMJ. 2021;372:n71. 33782057 10.1136/bmj.n71 PMC8005924

[17] York Uo. National Institute for Health Research. International Prospective Register of Systematic Reviews (PROSPERO). University of York. Accessed July 10, 2024. https://www.crd.york.ac.uk/prospero

[18] JBI. Analytical cross sectional studies. Accessed July 10, 2024. https://jbi.global/critical-appraisal-tools

[19] Chen D , Zhi Q, Zhou Y, Tao Y, Wu L, Lin H. Association between dental caries and BMI in children: a systematic review and meta-analysis. Caries Res. 2018;52:230-245. 10.1159/000484988 29353283

[20] Cepeda-Lopez AC , Osendarp SJ, Melse-Boonstra A, et al Sharply higher rates of iron deficiency in obese Mexican women and children are predicted by obesity-related inflammation rather than by differences in dietary iron intake. Am J Clin Nutr. 2011;93:975-983. 10.3945/ajcn.110.005439 21411619

[21] Clark P , Montiel-Ojeda D, Chico-Barba LG, López-González D, Méndez-Sánchez L, Guagnelli-Martínez MA. Vitamin D concentration and its association with parathyroid hormone in children and adolescents. Bol Med Hosp Infant Mex. 2021;78:265-272. 34107534 10.24875/BMHIM.20000243

[22] Denova-Gutiérrez E , Muñoz-Aguirre P, López D, et al Low serum vitamin D concentrations are associated with insulin resistance in Mexican children and adolescents. Nutrients. 2019;11:2109. 31491877 10.3390/nu11092109 PMC6770751

[23] Elizondo‐Montemayor L , Ugalde‐Casas PA, Serrano‐González M, Cuello‐García CA, Borbolla‐Escoboza JR. Serum 25‐hydroxyvitamin d concentration, life factors and obesity in Mexican children. Obesity. 2010;18:1805-1811. 20010726 10.1038/oby.2009.448

[24] Housni FE , Saenz-Pardo-Reyes E, de Jesús López Larios M, Cañedo CL, Cervantes VGA, Lares-Michel M. Association between nutritional status, deficiency of protein, iron and vitamins, caloric intake and food security in Mexi-can school children. Progr Nutr. 2022;24:2022013.

[25] Flores M , Macías N, Rivera M, et al Energy and nutrient intake among Mexican school-aged children, Mexican National Health and Nutrition Survey 2006. Salud Publica Mex. 2009;51(Suppl 4):S540-S550. 20464230 10.1590/s0036-36342009001000009

[26] Flores A , Flores M, Macias N, et al Vitamin D deficiency is common and is associated with overweight in Mexican children aged 1–11 years. Public Health Nutr. 2017;20:1807-1815. 28241892 10.1017/S1368980017000040 PMC10284713

[27] Flores Ruelas Y , Equihua MDT, Solís NAJ, Rodríguez LMB, Enciso ID, Ramírez CAS. Vitamin D status and its relation to insulin resistance in a Mexican pediatric population. J Pediatr Endocrinol Metab. 2020;33:481-486. 10.1515/jpem-2019-0510 32112703

[28] Flores ME , Rivera-Pasquel M, Valdez-Sánchez A, et al Vitamin D status in Mexican children 1 to 11 years of age: an update from the ENSANUT 2018-19. Salud Publ Mex. 2021;63:382-393. 10.21149/12156 34098608

[29] García OP , Ronquillo D, López LV, del Carmen Caamaño M, Rosado JL. Associations Between Plasma Micronutrients Concentrations With Overweight and Obesity in Children From Rural Mexico. Wiley Online Library; 2011.

[30] Martínez-Navarro I , Vilchis-Gil J, Cossío-Torres PE, et al Relationship of serum zinc levels with cardiometabolic traits in overweight and obese schoolchildren from Mexico City. Biol Trace Elem Res. 2023;201:4307-4319. 36572827 10.1007/s12011-022-03533-8 PMC9792317

[31] Ramos FR , Aradillas-García C, Díaz-Barriga F, Salas AP. Ingesta de macronutrientes y micronutrientes en adolescentes de una comunidad indígena de San Luis Potosí, México. Rev Esp Nutr Comun. 2013;19:152-158.

[32] Valdez López RM , Fausto Guerra J, Valadez FI, Ramos Ramos A, Loreto Garibay O, Villaseñor Farias M. Estado nutricional y carencias de micronutrientes en la dieta de adolescentes escolarizados de la zona metropolitana de Guadalajara, Jalisco [Nutritional state and shortcoming of micronutrients on schooled youth's diet on the metropolitan zone of Guadalajara Jalisco]. Arch Latinoam Nutr. 2012;62:161-166. 23610903

[33] Valle-Leal J , Limón-Armenta J, Serrano-Osuna R, López-Morales CM, Alvárez-Bastidas L. Forma activa de la vitamina D en pacientes pediátricos con sobrepeso y obesidad en el noroeste de México [Active form of vitamin D in overweight and obese pediatric patients in northwest Mexico]. Bol Med Hosp Infant Mex. 2017;74:413-418. 10.1016/j.bmhimx.2017.07.004 29382525

[34] Vargas-Hernández G , Romero-Velarde E, Vásquez-Garibay EM, Vizmanos-Lamotte B, Troyo-Sanromán R. Ingestión de calcio y adiposidad en adolescentes de 12 a 16 años en Guadalajara, México [Calcium intake and adiposity in adolescents aged 12-16 years in Guadalajara, Mexico]. Arch Latinoam Nutr. 2013;63:157-163. 24934072

[35] Cole TJ, , Bellizzi MC, , Flegal KM, , Dietz WH. Establishing a standard definition for child overweight and obesity worldwide: international survey. BMJ. 2000;320:1240-1243. 10.1136/bmj.320.7244.1240 10797032 PMC27365

[36] King JC , Brown KH, Gibson RS, et al Biomarkers of Nutrition for Development (BOND)—zinc review. J Nutr. 2015;146:858S-885S. 26962190 10.3945/jn.115.220079 PMC4807640

[37] Ciosek Ż , Kot K, Kosik-Bogacka D, Łanocha-Arendarczyk N, Rotter I. The effects of calcium, magnesium, phosphorus, fluoride, and lead on bone tissue. Biomolecules. 2021;11:506. 10.3390/biom11040506 PMC8066206 33800689

[38] Shlisky J , Mandlik R, Askari S, et al Calcium deficiency worldwide: prevalence of inadequate intakes and associated health outcomes. *Ann N Y Acad Sci*. 2022;1512:10-28. 10.1111/nyas.14758 PMC9311836 35247225

[39] Li P , Fan C, Lu Y, Qi K. Effects of calcium supplementation on body weight: a meta-analysis12. Am J Clin Nutr. 2016;104:1263-1273. 10.3945/ajcn.116.136242 27733391

[40] Dror DK , Allen LH. Vitamin E deficiency in developing countries. Food Nutr Bull. 2011;32:124-143. 22164974 10.1177/156482651103200206

[41] Ellulu MS. Obesity, cardiovascular disease, and role of vitamin C on inflammation: a review of facts and underlying mechanisms. Inflammopharmacology. 2017;25:313-328. 28168552 10.1007/s10787-017-0314-7

[42] Traber MG. Vitamin E inadequacy in humans: causes and consequences. Adv Nutr. 2014;5:503-514. 25469382 10.3945/an.114.006254 PMC4188222

[43] García OP , Ronquillo D, Del Carmen Caamaño M, et al Zinc, iron and vitamins A, C and E are associated with obesity, inflammation, lipid profile and insulin resistance in Mexican school-aged children. Nutrients. 2013;5:5012-5030. 24335710 10.3390/nu5125012 PMC3875915

[44] Hallberg L , Brune M, Rossander-Hulthén L. Is there a physiological role of vitamin C in iron absorption? Ann N Y Acad Sci. 1987;498:324-332. 10.1111/j.1749-6632.1987.tb23771.x 3304065

[45] Villalpando S , Montalvo-Velarde I, Zambrano N, et al Vitamins A, and C and folate status in Mexican children under 12 years and women 12-49 years: a probabilistic national survey. Salud Públ Méx. 2003;45(Suppl 4):508-519. 10.1590/s0036-36342003001000007 14746045

[46] Zhao L , Zhang X, Shen Y, Fang X, Wang Y, Wang F. Obesity and iron deficiency: a quantitative meta-analysis. Obes Rev. 2015;16:1081-1093. 10.1111/obr.12323 26395622

[47] Gibson RS , Raboy V, King JC. Implications of phytate in plant-based foods for iron and zinc bioavailability, setting dietary requirements, and formulating programs and policies. Nutr Rev. 2018;76:793-804. 10.1093/nutrit/nuy028 30010865

[48] Fiamenghi VI , Mello E. Vitamin D deficiency in children and adolescents with obesity: a meta-analysis. J Pediatr. 2021;97:273-279. 10.1016/j.jped.2020.08.006 PMC9432231 33022267

[49] Pérez-Bravo F , Duarte L, Arredondo-Olguín M, Iñiguez G, Castillo-Valenzuela O. Vitamin D status and obesity in children from Chile. Eur J Clin Nutr. 2022;76:899-901. 34773092 10.1038/s41430-021-01043-9 PMC9187513

[50] Plesner JL , Dahl M, Fonvig CE, et al Obesity is associated with vitamin D deficiency in Danish children and adolescents. J Pediatr Endocrinol Metab. 2018;31:53-61. 29197860 10.1515/jpem-2017-0246

[51] Turer CB , Lin H, Flores G. Prevalence of vitamin D deficiency among overweight and obese US children. Pediatrics. 2013;131:e152-e161. 10.1542/peds.2012-1711 23266927

[52] Florez H , Martinez R, Chacra W, Strickman-Stein N, Levis S. Outdoor exercise reduces the risk of hypovitaminosis D in the obese. J Steroid Biochem Mol Biol. 2007;103:679-681. 17267209 10.1016/j.jsbmb.2006.12.032

[53] Soltero EG , Jáuregui A, Hernandez E, et al Associations between screen-based activities, physical activity, and dietary habits in Mexican schoolchildren. Int J Environ Res Public Health. 2021;18:6788. 10.3390/ijerph18136788 34202680 PMC8297222

[54] Chen TC , Chimeh F, Lu Z, et al Factors that influence the cutaneous synthesis and dietary sources of vitamin D. Arch Biochem Biophys. 2007;460:213-217. 10.1016/j.abb.2006.12.017 17254541 PMC2698590

[55] Holick MF. Sunlight and vitamin D for bone health and prevention of autoimmune diseases, cancers, and cardiovascular disease. Am J Clin Nutr. 2004;80:1678s-1688s. 10.1093/ajcn/80.6.1678S 15585788

[56] Wortsman J , Matsuoka LY, Chen TC, Lu Z, Holick MF. Decreased bioavailability of vitamin D in obesity. Am J Clin Nutr. 2000;72:690-693. 10.1093/ajcn/72.3.690 10966885

[57] Charoenngam N , Shirvani A, Holick MF. Vitamin D for skeletal and non-skeletal health: what we should know. J Clin Orthop Trauma. 2019;10:1082-1093. 10.1016/j.jcot.2019.07.004 31708633 PMC6834997

[58] Holick MF. Resurrection of vitamin D deficiency and rickets. J Clin Invest. 2006;116:2062-2072. 10.1172/jci29449 16886050 PMC1523417

[59] Sahay M , Sahay R. Rickets-vitamin D deficiency and dependency. Indian J Endocrinol Metab. 2012;16:164-176. 10.4103/2230-8210.93732 22470851 PMC3313732

[60] Antonucci R , Locci C, Clemente MG, Chicconi E, Antonucci L. Vitamin D deficiency in childhood: old lessons and current challenges. J Pediatr Endocrinol Metab. 2018;31:247-260. 10.1515/jpem-2017-0391 29397388

[61] Maestro B , Dávila N, Carranza MC, Calle C. Identification of a vitamin D response element in the human insulin receptor gene promoter. J Steroid Biochem Mol Biol. 2003;84:223-230. 10.1016/s0960-0760(03)00032-3 12711007

[62] Chiu KC , Chu A, Go VL, Saad MF. Hypovitaminosis D is associated with insulin resistance and beta cell dysfunction. Am J Clin Nutr. 2004;79:820-825. 10.1093/ajcn/79.5.820 15113720

[63] DeLuca HF. Overview of general physiologic features and functions of vitamin D. Am J Clin Nutr. 2004;80:1689s-1696s. 10.1093/ajcn/80.6.1689S 15585789

[64] Charoenngam N , Holick MF. Immunologic effects of vitamin D on human health and disease. Nutrients. 2020;12:2097. 10.3390/nu12072097 PMC7400911 32679784

[65] da Silva L , da Veiga GV, Ramalho RA. Association of serum concentrations of retinol and carotenoids with overweight in children and adolescents. Nutrition. 2007;23:392-397. 17433621 10.1016/j.nut.2007.02.009

[66] Ford ES , Gillespie C, Ballew C, Sowell A, Mannino DM. Serum carotenoid concentrations in US children and adolescents. Am J Clin Nutr. 2002;76:818-827. 12324296 10.1093/ajcn/76.4.818

[67] Neuhouser ML , Rock CL, Eldridge AL, et al Serum concentrations of retinol, α-tocopherol and the carotenoids are influenced by diet, race and obesity in a sample of healthy adolescents. J Nutr. 2001;131:2184-2191. 11481415 10.1093/jn/131.8.2184

[68] Sarni ROS , de Souza FIS, Ramalho RA, et al Serum retinol and total carotene concentrations in obese pre-school children. Med Sci Monit. 2005;11:514. 16258394

[69] Langlois K , Cooper M, Colapinto CK. Vitamin C status of Canadian adults: findings from the 2012/2013 Canadian Health Measures Survey. Health Rep. 2016;27:3-10. 27192205

[70] Schleicher RL , Carroll MD, Ford ES, Lacher DA. Serum vitamin C and the prevalence of vitamin C deficiency in the United States: 2003–2004 National Health and Nutrition Examination Survey (NHANES). Am J Clin Nutr. 2009;90:1252-1263. 19675106 10.3945/ajcn.2008.27016

[71] Gaona-Pineda EB , Rodríguez-Ramírez S, Medina-Zacarías MC, Valenzuela-Bravo DG, Martinez-Tapia B, Arango-Angarita A. Consumidores de grupos de alimentos en población mexicana. ENSANUT Continua 2020-2022. Salud Públ México. 2023;65:s248-s258. 10.21149/14785 38060965

[72] El Laboratorio PROFECO. Alimentos adicionados con vitamina C. 2014. Accessed February 10, 2024. https://www.gob.mx/cms/uploads/attachment/file/100395/40-48RC443_Estudio_de_Calidad_VitaminaC.pdf

[73] Bird JK , Feskens EJ, Melse-Boonstra A. A systematized review of the relationship between obesity and vitamin C requirements. Curr Dev Nutr. 2024;8:102152. 10.1016/j.cdnut.2024.102152 38666038 PMC11039309

[74] Kreusler P , Vogel M, Willenberg A, et al Folate and cobalamin serum levels in healthy children and adolescents and their association with age, sex, BMI and socioeconomic status. Nutrients. 2021;13:546. 33562369 10.3390/nu13020546 PMC7915137

[75] Pinhas-Hamiel O , Doron-Panush N, Reichman B, Nitzan-Kaluski D, Shalitin S, Geva-Lerner L. Obese children and adolescents: a risk group for low vitamin B12 concentration. Arch Pediatr Adolesc Med. 2006;160:933-936. 16953016 10.1001/archpedi.160.9.933

[76] Sevim M , Abseyi SN. Overweight and obese adolescents: a risk group for vitamin B12 deficiency and anemia? J Surg Med. 2022;6:391-394.

[77] Xanthakos SA. Nutritional deficiencies in obesity and after bariatric surgery. Pediatr Clin. 2009;56:1105-1121. 10.1016/j.pcl.2009.07.002 PMC2784422 19931066

[78] Santiago-Arango M , Pérez-Campos E, Porras-Chaparro I, et al Agreement on the prevalence of body mass index (BMI) in Mexican children and adolescents using different international references. Nutrients. 2025;17:587. 39940445 10.3390/nu17030587 PMC11819738

[79] Wells JC , Sawaya AL, Wibaek R, et al The double burden of malnutrition: aetiological pathways and consequences for health. Lancet. 2020;395:75-88. 10.1016/S0140-6736(19)32472-9 31852605 PMC7613491

