# Supplementary material for: Obesity and Its Association With Micronutrient Deficiency Among Mexican Children and Adolescents: A Systematic Review and Meta-analysis
Source: Nutr Rev. 2025 Aug 13;84(7):1309–24. doi: 10.1093/nutrit/nuaf148 (PMC13250450; doi:10.1093/nutrit/nuaf148)
Supplement: nuaf148_Supplementary_Data [file nuaf148_supplementary_data.docx]

**APPENDIX 1. Search Strategy**

Embase and MEDLINE(R)

| 1 | child/ or juvenile/ or boy/ or girl/ or infant/ or exp preschool child/ or exp school child/ or  toddler/ |
| --- | --- |
| 2 | (Child* or Kids or Infan* or Toddlers or Preschoolers or Schoolers or High schoolers or  Teenagers or Youth or Youngster or Juveniles or Minors or Adolescence or Adolescent or p?ediatric).ab,kf,ti. |
| 3 | 1 or 2 |
| 4 | obesity/ or overnutrition/ or abdominal obesity/ or adolescent obesity/ or childhood  obesity/ |
| 5 | body mass index/ |
| 6 | (Obes* or Overweight or Excess weight or Body weight or BMI or Body mass index or  Weight gain or Body composition or Waist Circumference or Waist-hip ratio or Electric impedance or Skinfold thickness or Adiposity or Fatness or Body fat or Fat percentage).ab,kf,ti. |
| 7 | 4 or 5 or 6 |
| 8 | exp nutritional deficiency/ or exp mineral deficiency/ or exp vitamin deficiency/ |
| 9 | (Vitamins or minerals or Micronutrient deficien* or Malnutrition or Malnouris* or Nutr*  deficien* or Undernutrition or Nutrient inadequacy or Mineral deficien* or Trace element deficiency or Iron deficiency or Hypoferritin?emia or an?emi* or Iodine deficiency or Zinc deficiency or Calcium deficiency or Hypocalc?emia or Potassium deficiency or Hypokal?emia or Magnesium deficiency or Hypomagnes?emia or Vitamin deficien* or (vitamin adj20 deficien*) or Thiamine deficiency or Riboflavin deficiency or Niacin deficiency or Pantothenic acid deficiency or Pyridoxine deficiency or Biotin deficiency or Folic acid deficiency or Folate deficiency or Cobalamin deficiency or Ascorbic acid deficiency or Cholecalciferol deficiency or Calcitriol deficiency or 25-hydroxy vitamin D deficiency or Tocopherol deficiency).ab,kf,ti |
| 10 | 8 or 9 |
| 11 | exp Mexico/ |
| 12 | (Mexico or Mexica* or Mexican republic or United Mexican States).ab,kf,ti. |
| 13 | 11 or 12 |
| 14 | 3 and 7 and 10 and 13 |
| 15 | remove duplicates from 14 |

*Any further search was adapted from this search strategy.*
